# Neutrophil proteases are protective against SARS-CoV-2 by degrading the spike protein and dampening virus-mediated inflammation

**DOI:** 10.1172/jci.insight.174133

**Published:** 2024-03-12

**Authors:** Nathan G.F. Leborgne, Christelle Devisme, Nedim Kozarac, Inês Berenguer Veiga, Nadine Ebert, Aurélie Godel, Llorenç Grau-Roma, Melanie Scherer, Philippe Plattet, Volker Thiel, Gert Zimmer, Adriano Taddeo, Charaf Benarafa

**Affiliations:** 1 Institute of Virology and Immunology, Mittelhäusern, Switzerland.; 2 Department of Infectious Diseases and Pathobiology, Vetsuisse Faculty,; 3 Graduate School for Cellular and Biomedical Sciences,; 4 COMPATH, Institute of Animal Pathology, Vetsuisse Faculty,; 5 Division of Neurological Sciences, Vetsuisse Faculty, and; 6 Multidisciplinary Center for Infectious Diseases (MCID), University of Bern, Bern, Switzerland.

**Keywords:** COVID-19, Inflammation, Neutrophils, Proteases, Serpins

## Abstract

Studies on severe acute respiratory syndrome coronavirus type 2 (SARS-CoV-2) have highlighted the crucial role of host proteases for viral replication and the immune response. The serine proteases furin and TMPRSS2 and lysosomal cysteine proteases facilitate viral entry by limited proteolytic processing of the spike (S) protein. While neutrophils are recruited to the lungs during COVID-19 pneumonia, little is known about the role of the neutrophil serine proteases (NSPs) cathepsin G (CatG), elastase (NE), and proteinase 3 (PR3) on SARS-CoV-2 entry and replication. Furthermore, the current paradigm is that NSPs may contribute to the pathogenesis of severe COVID-19. Here, we show that these proteases cleaved the S protein at multiple sites and abrogated viral entry and replication in vitro. In mouse models, CatG significantly inhibited viral replication in the lung. Importantly, lung inflammation and pathology were increased in mice deficient in NE and/or CatG. These results reveal that NSPs contribute to innate defenses against SARS-CoV-2 infection via proteolytic inactivation of the S protein and that NE and CatG limit lung inflammation in vivo. We conclude that therapeutic interventions aiming to reduce the activity of NSPs may interfere with viral clearance and inflammation in COVID-19 patients.

## Abstract 2

Neutrophil granule serine proteases cleave the spike protein and are mainly protective in early SARS-CoV-2 infection in mice.

## Graphical abstract

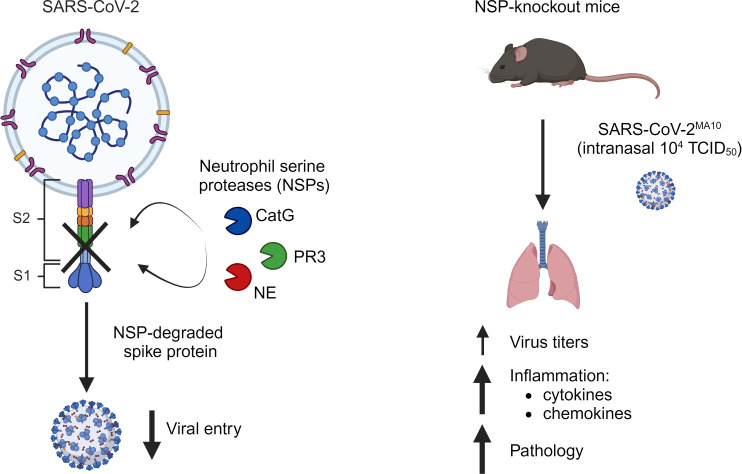

## Introduction

SARS-CoV-2 is a zoonotic β-coronavirus and the cause of the COVID-19 pandemic (1). The viral spike (S) protein is essential for SARS-CoV-2 attachment and entry into host cells, making it an ideal target for vaccination and antivirals (2). The S protein is a type I transmembrane glycoprotein, with a size of 180–200 kDa present in a trimeric form at the viral envelope. It is composed of an N-terminal signal peptide and 2 subunits, S1 and S2, that have distinct functions for viral entry. The S1 subunit contains the receptor-binding domain (RBD), which mediates the attachment of virions to the plasma membrane through high-affinity interaction with the host cell surface receptor angiotensin-converting enzyme 2 (ACE2) (3). The S2 subunit contains the fusion peptide that mediates the fusion of the viral envelope with cellular membranes. Proteolytic maturation of S into the S1 and S2 subunits by host proteases induces conformational changes that increase affinity for ACE2 and drive membrane fusion and viral entry. In contrast with closely related coronaviruses, SARS-CoV-2 S contains a polybasic amino acid insertion at the S1-S2 junction that is cleaved by proprotein convertases such as furin in the Golgi apparatus during viral biosynthesis and maturation (4, 5). Released virions then require further processing of the S2 subunit by transmembrane protease serine 2 (TMPRSS2) at the plasma membrane, or by the cysteine protease cathepsin L in the endosomal pathway (6–8). Pharmacological inhibition and genetic deletion of TMPRSS2 have demonstrated that it is a key factor promoting entry of all SARS-CoV-2 variants of concern in preclinical mouse models (9, 10). A disintegrin and metalloproteinase 10 (ADAM10) and ADAM17 were also shown to contribute to a lesser extent to S processing and SARS-CoV-2 entry (11, 12).

Virus-specific prophylactic and therapeutic measures such as vaccines and neutralizing monoclonal antibodies against the S protein have shown remarkable efficacy in reducing hospitalization and death (2, 13). The inhibition of inflammatory reactions improved disease symptoms for mild to critical illness. In hospitalized COVID-19 patients, systemic corticosteroids were a crucial therapy associated with improved recovery and survival (14). Antiviral drugs targeting viral structural proteins and enzymes were shown to be effective at preventing the development of acute respiratory distress syndrome (ARDS) when given in the early phase of infection (2). However, their efficacy is subject to immune evasion and acquired resistance through selection. Thus, the development of drugs targeting the host mechanisms involved in the virus life cycle, such as host proteases and receptors involved in attachment and entry into target cells, is a complementary approach that may be effective against SARS-CoV-2 and also a spectrum of related viruses (15).

Neutrophil serine proteases (NSPs) are key components of innate defences against microbes. They are synthesized in the early stages of granulopoiesis and stored within primary granules, which are specialized lysosomes. The broad antimicrobial cargo of primary granules of neutrophils is released into phagosomes to digest their contents or, in the extracellular milieu following strong degranulation stimuli (16). The main NSPs include cathepsin G (CatG), neutrophil elastase (NE), and proteinase-3 (PR3), which help kill phagocytosed bacteria and fungi (17–19). More critically, NSPs regulate the activity and release of cytokines, chemokines, growth factors, and cell surface receptors with inhibitory and enhancing effects on inflammation (20–26). In the lung, unregulated NSPs are commonly thought to drive excessive inflammation and extracellular matrix destruction in acute and chronic lung diseases (27, 28). The role of NSPs in SARS-CoV-2 entry and the severity of lung disease of COVID-19 patients remains undefined. In silico studies and experimental data based on small peptide cleavage suggest that NSPs can process SARS-CoV-2 S protein at the S1/S2 boundary; however, these studies did not investigate cleavage of full-length S protein and the resulting effect on viral entry is unknown (29–32). Moreover, as active NSPs have been found in the lung of COVID-19 patients with ARDS, the use of NSP inhibitors, such as α1-antitrypsin (α1AT), has been proposed in COVID-19 patients and particularly in patients with cystic fibrosis or α1AT deficiency (33–36). Here, we examined the proteolysis of SARS-CoV-2 S protein by CatG, NE, and PR3, and viral entry in vitro. We then tested the effect of these proteases on SARS-CoV-2 viral load, inflammation, and lung pathology. Overall, our results demonstrate that NSPs, particularly CatG and NE, are important antiviral molecules that protect the host against viral entry and proliferation, and prevent excessive virus-mediated inflammation.

## Results

### Efficient proteolytic processing of SARS-CoV-2 S by NSPs.

**Figure 1:**
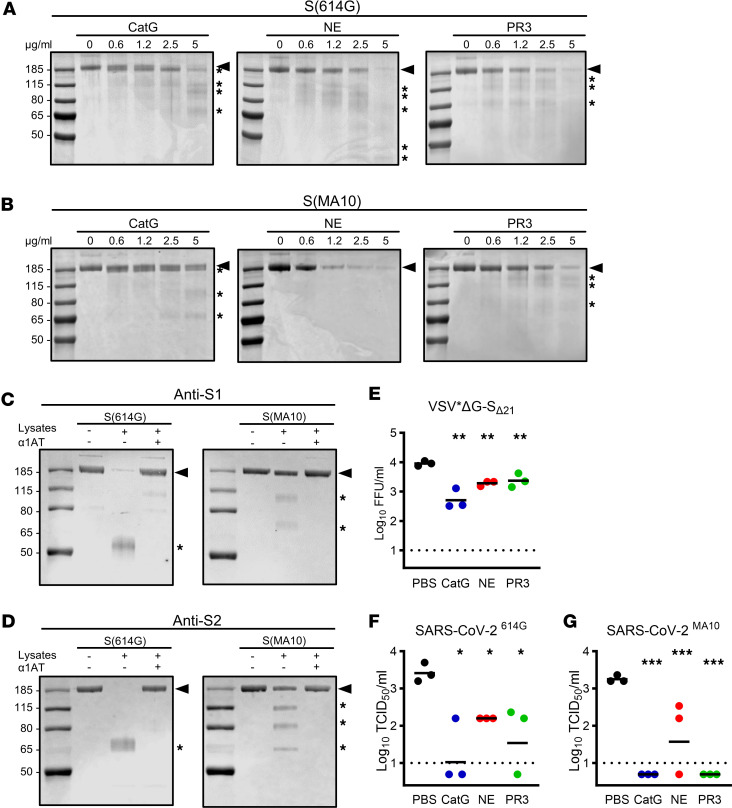
Neutrophil serine proteases degrade S and inhibit SARS-CoV-2 entry. (**A** and **B**) SDS-PAGE of recombinant trimeric S(614G) (**A**) and S(MA10) (**B**) incubated with purified human CatG, NE, and PR3 at indicated concentrations. Arrowheads indicate S protein and asterisks cleaved fragments. (**C** and **D**) Immunoblots of recombinant trimeric S(614G) and S(MA10) incubated with mouse neutrophil lysates with or without α1AT and probed with anti-S1 (**C**) or anti-S2 (**D**) subunit antibodies. (**E**) Titers of VSV*ΔG-S_Δ21_ incubated with NSPs. (**F** and **G**) Titers of SARS-CoV-2^614G^ (**F**) and SARS-CoV-2^MA10^ (**G**) incubated with NSPs. Data in **A**–**D** representative of at least 3 independent experiments. Data in **E**–**G** were analyzed by 1-way ANOVA with Dunnett’s multiple-comparison test, comparing the NSP-treated group to the PBS control (*n* = 3). *P < 0.05, **P < 0.01, ***P < 0.001.

We first investigated the proteolytic processing of recombinant soluble trimeric SARS-CoV-2 S protein by NSPs in vitro. Purified human CatG, NE, and PR3 efficiently cleaved the ancestral B.1 lineage S(614G) in a dose-dependent manner (Figure 1A). CatG produced a distinct proteolytic pattern, with 2 products at 175 and 100 kDa at low CatG concentration, and 2 more stable bands at 90 and 65 kDa appearing at higher CatG concentrations, suggesting successive proteolytic processing. NE and PR3 appeared to degrade the full-length S(614G) (185 kDa), with small amounts of low molecular weight cleavage products detectable. We then used trimeric S(MA10) containing mutations (Q498Y/P499T/Q493K) that permit binding of S to mouse ACE2. CatG generated a similar pattern of stable products with S(MA10), with 175, 90, and 65 kDa fragments except for the 100 kDa fragment, which was only observed with S(614G). PR3 also produced stable proteolytic 145, 130, and 75 kDa fragments of S(MA10), whereas NE degraded S(MA10) without detectable stable proteolytic fragments (Figure 1B). To test the effects of mouse NSPs on SARS-CoV-2 S processing, lysates of mouse neutrophils containing active NSPs were incubated with WT S(614G) and mouse-adapted S(MA10). Western blot analysis revealed a similar cleavage of S(614G) and S(MA10) by mouse NSPs, with fragments at 100 and 65 kDa (Figure 1, C and D). The cleavage of S was inhibited by α1AT, a specific inhibitor of the 3 NSPs, indicating that S proteolysis was mediated by NSPs and not by other neutrophil proteases.

### NSPs impair pseudovirus entry in vitro.

To better mimic S exposure on a virion and to determine the consequences of S proteolysis on viral entry, we used a chimeric vesicular stomatitis virus (VSV) expressing S(614G) in place of the VSV surface glycoprotein G (VSV*ΔG-S_Δ21_), in which the S protein lacks 21 amino acids at the cytosolic C-terminal tail. Preincubation of VSV*ΔG-S_Δ21_ (1 × 10^4^ focus-forming units [FFU]) with 5 μg/mL (170 nM) NE, CatG, or PR3 for 4 hours significantly reduced viral titers by approximately 1 order of magnitude (Figure 1E). When higher and lower FFU of VSV*ΔG-S_Δ21_ (4 × 10^3^ to 4 × 10^4^ FFU) were preincubated with serial dilutions of NSPs (0.6–5 μg/mL), we found that reduction of VSV*ΔG-S_Δ21_ entry correlated with the ratio of protease to substrate. Furthermore, we did not observe an increase in FFU when VSV*ΔG-S_Δ21_ was preincubated with lower NSP concentrations (Supplemental Figure 1A; supplemental material available online with this article; https://doi.org/10.1172/jci.insight.174133DS1). In these experiments, NSP-treated VSV*ΔG-S_Δ21_ was added to Vero/TMPRSS2 cells in the presence of 10% fetal bovine serum (FBS), where serpins are in sufficient concentration to almost completely abolish NSP activity (Supplemental Figure 1B). To further investigate the possibility that NSPs could also inhibit viral entry through cleavage or degradation of cell surface proteins, NSPs (5 μg/mL) were incubated for 4 hours with Vero/TMPRSS2 cells in the absence of FBS, and then washed with medium containing FBS and inoculated with VSV*ΔG-S_Δ21_. A modest, but statistically significant, reduction in VSV*ΔG-S_Δ21_ entry was observed in cells preincubated with CatG, but not with NE or PR3, prior to infection (Supplemental Figure 1C). Finally, VSV*ΔG-S_Δ21_ entry was not altered by NSPs after it was adsorbed to Vero/TMPRSS2 cells for 30 minutes before adding the NSPs (Supplemental Figure 1D). Together, these data indicate that inhibition of VSV*ΔG-S_Δ21_ entry in vitro is principally mediated by the degradation of S by the 3 NSPs, and that NSPs did not enhance viral entry through activating proteolysis of S, although full-length S is highly abundant on the surface of VSV*ΔG-S_Δ21_ (Supplemental Figure 1E).

### NSPs impair SARS-CoV-2 entry in vitro.

To assess the effects of NSPs directly on SARS-CoV-2 entry and replication, SARS-CoV-2^614G^ was incubated with NSPs for 4 hours and viral titers subsequently determined on Vero E6 cells. Remarkably, all 3 NSPs significantly reduced infectious virus titers (Figure 1F). We also used SARS-CoV-2^MA10^, which was originally generated by passaging recombinant SARS-CoV-2 encoding the S(Q498Y/P499T) mutant protein (37) in mouse lungs to acquire 1 further mutation (Q493K) in the S protein and 4 mutations in nonstructural protein-4, -7, and -8, and open reading frame 6 (38). The SARS-CoV-2^MA10^ titer was also significantly reduced by the 3 proteases (Figure 1G). These results suggest that CatG, NE, and PR3 reduce or even abrogate SARS-CoV-2 entry and replication by proteolytic inactivation of the S protein.

### CatG reduces SARS-CoV-2 titers in the lungs.

**Figure 2:**
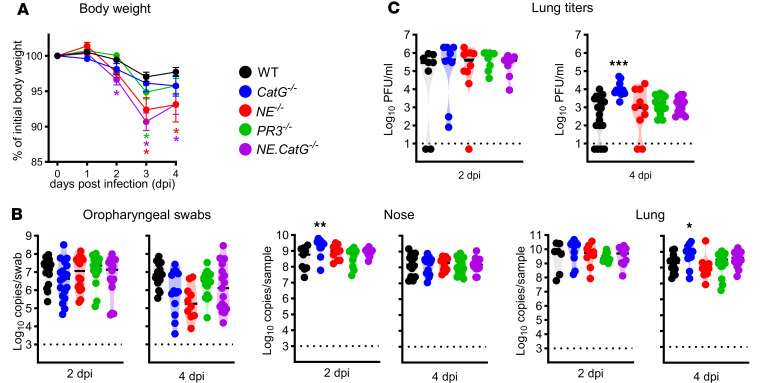
*CatG^–/–^* mice have increased viral replication in the lungs and in the nose compared with WT mice. WT, *CatG^–/–^*, *NE^–/–^*, *PR3^–/–^*, and *NE.CatG^–/–^* mice were inoculated intranasally with 1 × 10^4^ TCID_50_ SARS-CoV-2^MA10^. (**A**) Relative body weight change overtime after infection. Data were analyzed by 2-way ANOVA with Dunett’s multiple-comparison test, comparing the knockout groups to the WT group. Data were pooled from 6 independent experiments (*n* = 12–31 mice/group). *P < 0.05; color of asterisk corresponds to the genotype. (**B**) Viral copy numbers were determined by RT-qPCR in oropharyngeal swabs or tissue homogenates. (**C**) Viral titers in lung homogenates. (**B** and **C**) Data were log_10_ transformed and analyzed by 1-way ANOVA with Dunnett’s multiple-comparison test comparing the knockout groups to the WT group at each time point. Data pooled from 7 independent experiments (2 dpi, *n* = 16–19/group; 4 dpi, *n* = 10–17/group). *P < 0.05, **P < 0.01, ***P < 0.001.

To investigate the effects of the NSPs on SARS-CoV-2 replication in vivo, mice deficient for each NSP (*CatG^–/–^*, *NE^–/–^*, *PR3^–/–^*) and double-deficient *NE.CatG^–/–^* mice were inoculated intranasally with 1 × 10^4^ TCID_50_ of SARS-CoV-2^MA10^. *NE^–/–^* and *PR3^–/–^* mice showed decreased body weight compared with WT mice at 3 days post infection (dpi), while *NE.CatG^–/–^* mice had decreased body weight on 2–4 dpi (Figure 2A). The viral copy numbers were similar between all genotypes in the oropharyngeal swabs at 2 and 4 dpi. Viral copies were significantly increased in the nose (2 dpi) and lungs (4 dpi) of *CatG^–/–^* mice (Figure 2B). Similarly, viral titers were significantly increased in lungs of *CatG^–/–^* compared with WT mice at 4 dpi (Figure 2C). In contrast, viral loads and titers in noses and lungs of *NE^–/–^*, *PR3^–/–^*, and *NE.CatG^–/–^* mice were not different from those of WT mice (Figure 2, B and C). These results indicate that CatG inhibits SARS-CoV-2^MA10^ replication in vivo and that the NSPs contribute alone or in combination to enhance pathologic effects by SARS-CoV-2^MA10^, as evident from loss of body weight.

### Increased lung inflammation in NSP-deficient mice infected with SARS-CoV-2.

**Figure 3:**
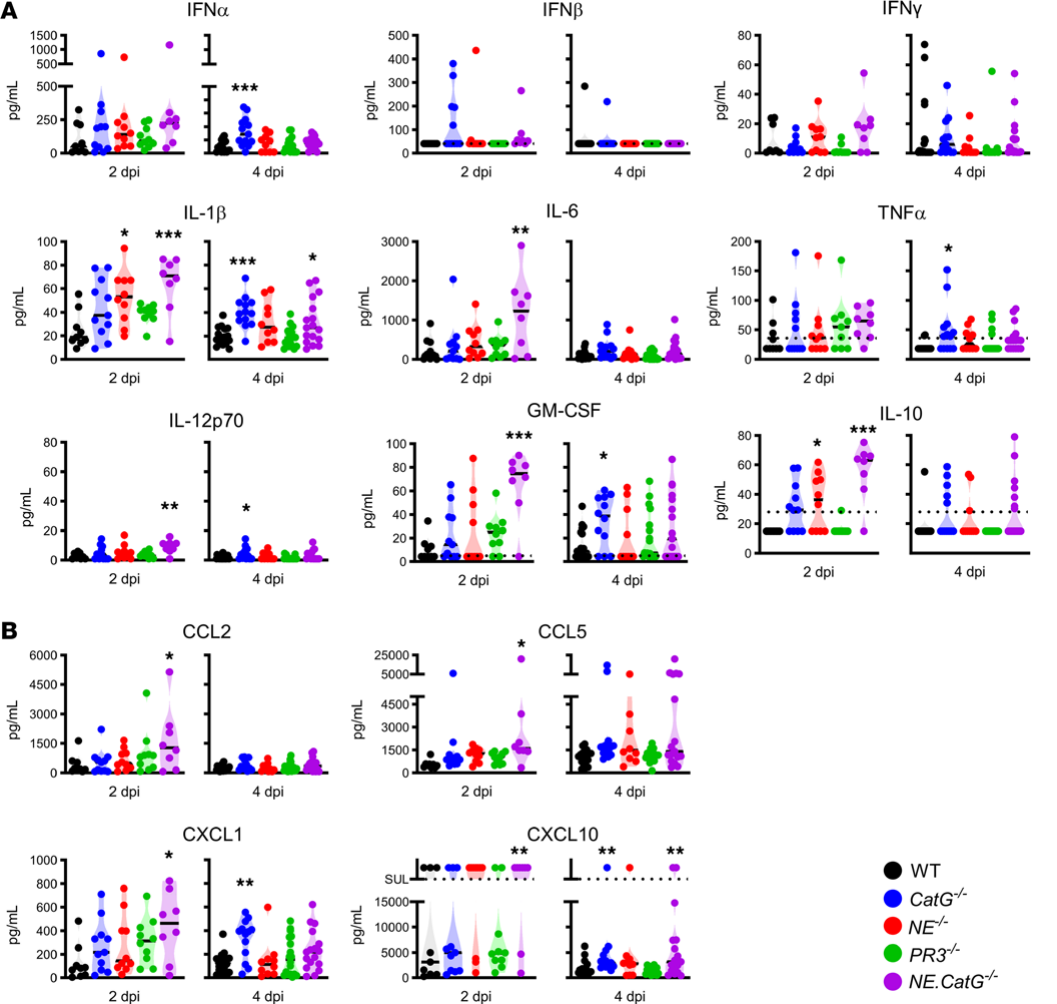
NE and CatG regulate levels of cytokines and chemokines in the lungs. (**A** and **B**) WT, *CatG^–/–^*, *NE^–/–^*, *PR3^–/–^*, and *NE.CatG^–/–^* mice were inoculated intranasally with 1 × 10^4^ TCID_50_ SARS-CoV-2^MA10^. Cytokines (**A**) and chemokines (**B**) were measured in lung homogenates on the indicated dpi. Data were analyzed by 1-way ANOVA with Dunnett’s multiple-comparison test comparing the groups of knockout mice to the WT group at each time point. Data pooled from 7 independent experiments (2 dpi, *n* = 8–11/group; 4dpi, *n* = 10–19/group). *P < 0.05, **P < 0.01, ***P < 0.001.

**Figure 4:**
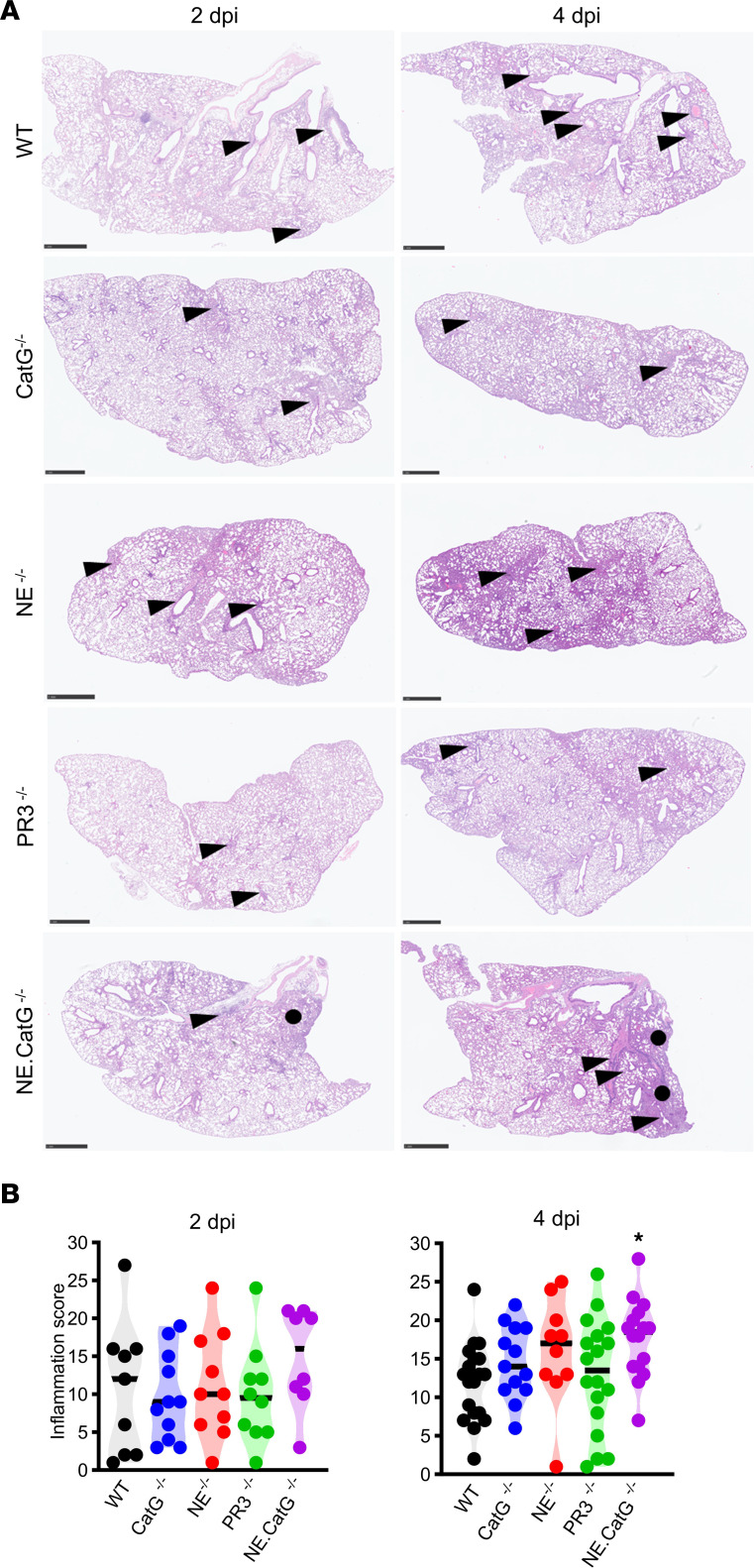
Increased acute pathological lesions in *NE.CatG^–/–^* mice after SARS-CoV-2 infection. (**A**) Representative histopathological lesions in the lungs of mice inoculated with 1 × 10^4^ TCID_50_ SARS-CoV-2^MA10^ at the indicated dpi. The black dots indicate consolidated areas corresponding to interstitial pneumonia, and the arrowheads indicate peribronchiolar and perivascular mononuclear infiltrates. H&E staining. Scale bars: 1 mm. (**B**) Lung inflammation score was analyzed by 1-way ANOVA with Dunnett’s multiple-comparison test comparing the knockout groups to the WT group. *P < 0.05. Data were pooled from 7 independent experiments (2 dpi, *n* = 8–11/group; 4 dpi, *n* = 10–19/group).

To further explore the effect of NSPs in the severity of SARS-CoV-2^MA10^–induced ARDS, we measured the cytokine levels in lung homogenates of infected mice at 2 and 4 dpi. Type I interferon (IFN-α) was significantly increased in *CatG^–/–^* mice compared with WT mice (Figure 3A), correlating with the increased viral titers in these mice (Figure 2C). Proinflammatory cytokines (IL-1β, TNF, IL-6, IL-12p70) and GM-CSF were generally increased in *NE^–/–^*, *CatG^–/–^*, and/or *NE*.*CatG^–/–^* mice compared with WT mice (Figure 3A). In contrast, no significant changes were observed in *PR3^–/–^* mice compared to WT controls. Chemokines important for recruitment of neutrophils (CXCL1), monocytes (CCL2, CCL5), and T cells (CCL5, CXCL10) were also significantly elevated in *CatG^–/–^* and particularly in *NE.CatG^–/–^* mice (Figure 3B). These results suggest that the body weight loss observed in *NE.CatG^–/–^* mice may be due to direct or indirect inhibition of the inflammatory cytokine and chemokine response by NE and CatG after SARS-CoV-2 infection (Figure 2A). Histopathological analysis of the lungs of infected mice revealed lesions characterized by multifocal necrosis of the bronchiolar epithelium and accumulation of cellular debris admixed with macrophages in the lumen of bronchioli and alveoli at 2 dpi, which evolved to moderate to severe peribronchiolar, perivascular, and interstitial mononuclear infiltration consistent with bronchointerstitial pneumonia at 4 dpi (Figure 4A). *NE.CatG^–/–^* mice had significantly more severe lesions than WT mice at 4 dpi (Figure 4B), which is consistent with the increase in molecular markers of inflammation in *NE.CatG^–/–^* mice.

## Discussion

Our findings demonstrate that NSPs cleave SARS-CoV-2 S protein and impair the early steps of the virus cycle in vitro. Previous reports based on the cleavage of short peptides of the S protein of different SARS-CoV-2 variants previously suggested that NSPs could cleave the S protein and speculated that such cleavage may support S activation and could contribute to disease severity (29–32). The present work shows that trimeric recombinant S is cleaved at multiple sites and eventually degraded by NSPs. We further showed that cleavage by NSPs inhibited entry of chimeric VSV*ΔG-S_Δ21_ pseudovirus expressing the S protein and that NSPs significantly reduced SARS-CoV-2 entry and replication in vitro. Using lower concentrations of NSPs or pseudovirus adsorbed to cells, we did not observe enhancement of viral entry. NSP concentrations used are comparable to those found in the lungs of patients with ARDS (36), where NSPs can reach 100 ng/mL in samples highly diluted (up to 100-fold) by the procedure of endotracheal aspirates or bronchoalveolar lavage. In sputum of cystic fibrosis patients, NE was reported at 30 μg/mL as a free, active enzyme (39). Furthermore, NSP activity was reported at high levels at the neutrophil surface, a compartment that is often overlooked (40). Therefore, the NSP concentrations used are appropriate when studying neutrophil-dominated lung diseases.

It is also likely that the NSPs act synergistically to degrade the S protein, as shown for many of their substrates, including extracellular matrix proteins, surfactant proteins, immunoglobulins, and soluble and membrane-bound inflammatory ligands and receptors (25, 41). Interestingly, we found that the degradation of the S protein by mouse neutrophil lysates was fully inhibited by an excess of α1AT, which inhibits NE, CatG, and PR3, but not other neutrophil proteases such as NSP4, matrix metalloproteases, cysteine proteases, and caspases. This suggests that the main proteases of neutrophils that proteolytically degrade the S protein of SARS-CoV-2 are these 3 serine proteases. A recent study showed that NE cleaved the N-terminal ectodomain of recombinant ACE2, which interfered with the binding of recombinant trimeric S to ACE2 in vitro (42). The effect of CatG and PR3 on ACE2 was not reported. Cleavage of ACE2 by NE suggests an additional antiviral mechanism for NE, although this was not directly investigated (42). Our in vitro data suggest that the blockade of VSV*ΔG-S_Δ21_ entry is principally mediated by the proteolytic degradation of S by the 3 NSPs. In our assay conditions using Vero/TMPRSS2 cells, the effects of NE and PR3 did not reach the required threshold to reduce viral entry, whereas CatG partially inhibited VSV*ΔG-S_Δ21_ entry. It remains to be tested whether CatG also cleaves ACE2 or acts through a different mechanism. The cleavage of ACE2 by NE, and potentially other proteases, may also have consequences for inflammatory responses through the regulation of the renin-angiotensin system (43).

The functions of NSPs in the context of viral infections is complex and insufficiently studied; reports have shown both enhancing or inhibiting effects of NSPs on viral entry and replication (44, 45). Using knockout mice, we demonstrated that NSPs participate to different degrees in reducing SARS-CoV-2 replication and inflammation in vivo. Lack of NE and PR3 had a direct impact on clinical signs associated with infection with SARS-CoV-2^MA10^, as *NE^–/–^* and *PR3^–/–^* mice lost significantly more body weight than WT mice after infection. *CatG^–/–^* mice showed significantly higher viral titers in the lungs compared with WT mice. *NE.CatG^–/–^* mice were the most severely affected in terms of body weight loss, increased inflammation, and lung histopathology. Surprisingly, viral titers in the lungs of *NE*.*CatG^–/–^* mice were not increased, as was observed in *CatG^–/–^* mice, suggesting that increased inflammation in *NE.CatG^–/–^* mice may have a double-edged effect causing more pathology, while helping other mechanisms of virus control. How the NSPs reduce cytokine and chemokine levels and lung pathology remains to be specifically elucidated and is likely combinatorial and not simply additive. On the one hand, S degradation by NSPs reduces viral entry and therefore alters the extent of subsequent IFN-induced responses such as the production of high levels of CXCL10. On the other hand, the NSPs were shown to directly inactivate cytokines, chemokines, and receptors through proteolysis (25, 46, 47). These data indicate multiple functions for each NSP and that the NSPs not only contribute to virus control but also lead to reduced inflammation and lung damage. The antiviral functions of NSPs against SARS-CoV-2 are likely to be influenced by the kinetics of infection and the presence of additional factors that alter neutrophil recruitment, activation, and degranulation.

Several clinical trials using α1AT therapy were initiated on the premise that NSP activity should be curbed, as it may play a role in the pathogenesis of severe COVID-19. Indeed, α1AT is the most abundant inhibitor of NSPs in plasma and α1AT deficiency (AATD) is the most important genetic predisposition to emphysema and chronic obstructive pulmonary disease, where proteases drive the pathology. Moreover, α1AT binds to TMPRSS2 at high concentrations, impairing S processing by TMPRSS2 and preventing SARS-CoV-2 entry to some extent (48–51). Our results, however, challenge the notion that reduction of NSP activity is a critical target in severe COVID-19 patients and our data are supported by recent epidemiological and clinical trial results. Indeed, in contrast with initial fears at the start of the pandemic, very large cohort studies and genome-wide association studies have shown that AATD is not associated with increased risk of a severe course of COVID-19 (52–54). In a large study of AATD patients, poor outcome of COVID-19 was more associated with nonrespiratory comorbidities than with genotype or respiratory function tests (55). Moreover, published clinical trials showed that intravenous α1AT or inhibition of cathepsin C, a dipeptidyl peptidase required for the maturation of NSPs into active enzymes, did not improve the clinical course of hospitalized COVID-19 patients (56, 57). In conclusion, the use of NSP inhibitors in COVID-19 patients and interpretation of clinical trial data should be weighed and interpreted in the light of the antiviral and antiinflammatory effects of NSPs in SARS-CoV-2 infection shown here.

## Methods

### Sex as a biological variable.

Our study examined male and female mice, and similar findings are reported for both sexes.

### Cells.

Vero E6 cells (Vero C1008, ATCC) or Vero cells expressing TMPRSS2 (Vero/TMPRSS2) (NIBSC Research Reagent Depository) were cultured at 37°C and 5% CO_2_ in Dulbecco’s modified Eagle medium with GlutaMAX (DMEM) (Thermo Fisher Scientific) supplemented with 1 mM sodium pyruvate, 10% (v/v) FBS, 100 μg/mL streptomycin, 100 IU/mL penicillin, and nonessential amino acids (Vero/TMPRSS2 only).

### Viruses.

All experiments with infectious SARS-CoV-2 were performed in biosafety level 3 (BSL3) containment laboratories. Infectious cDNA clones were generated using an in-yeast transformation–associated recombination cloning method, as previously described (58). The sequence of recombinant SARS-CoV-2 encoding the S(D614G) substitution (SARS-CoV-2^614G^) was published previously (59). The mouse-adapted 10 (MA10) SARS-CoV-2 cDNA clone was based on the previously published SARS-CoV-2^MA10^ sequence (38), including the D614G/Q498Y/P499T/Q493K substitutions in the S protein. In vitro transcription was performed for *Eag*I-cleaved YAC using the T7 RiboMAX Large Scale RNA production system (Promega). Transcribed capped mRNA was electroporated into baby hamster kidney cells (BHK-21 cells) expressing SARS-CoV-2 N protein. Electroporated cells were cocultured with susceptible Vero/TMPRSS2 cells to produce passage 0 of recombinant SARS-CoV-2. Progeny viruses were used to infect Vero/TMPRSS2 cells to generate passage 1 viral stocks for downstream experiments. Stock virus sequences were verified by whole-genome next-generation sequencing and titers were determined by TCID_50_ assay.

### TCID_50_ assay.

Vero E6 cells were seeded in 96-well plates using 2 × 10^4^ cells per well. One day after seeding, cells were inoculated with 10-fold serial dilutions of virus and incubated for 72 hours at 37°C and 5% CO_2_. Four technical replicates were performed for each sample. After incubation, inoculum was removed and cells were fixed with neutral-buffered formalin (4% v/v) and stained with crystal violet. Infected wells were counted manually and TCID_50_ was calculated according to the Spearman-Karber method.

### Trimeric S protein cleavage assay.

Recombinant trimeric S(614G) or S(MA10) proteins with additional proline for stabilization and deleted furin cleavage site were prepared as described previously (60). Neutrophils were isolated from the bone marrow of C57BL/6J mice. Briefly, tibia, femur, and iliac bones were flushed with 10 mL cold PBS without cations supplemented with 1% FBS and 1% pen/strep. Cell suspensions were filtered through a 70-μm cell strainer and red blood cells were lysed using ammonium chloride for 5 minutes at room temperature. Neutrophils were then purified by positive selection using anti-Ly6G beads and LS columns (Miltenyi Biotec). Purified neutrophils were lysed at 1 × 10^7^ cells/mL by repeated pipetting in cold NP-40 buffer (0.05% Nonidet P-40 substitute, 50 mM HEPES, 0.75 M NaCl) (61) followed by vortexing twice for 30 seconds with 2-minute resting on ice between cycles. Lysates were clarified by centrifugation at 18,000*g* for 10 minutes at 4°C. For in vitro cleavage, 1 μg of trimeric S was incubated with purified human CatG, NE, and PR3 (Athens Research Technology) at indicated final concentrations in 20 μL reactions for 1 hour at 37°C. Similarly, 0.5 μg of trimeric S was incubated with 50 μg neutrophil lysates with or without 250 ng α1AT (Athens Research Technology). Reactions were stopped by addition of 2× Laemmli buffer containing DTT and subsequent boiling for 5 minutes. S proteins were resolved in 10% Tris-glycine acrylamide gels (GenScript). Gels were stained overnight with QC Colloidal Coomassie (Bio-Rad) at room temperature and destained in deionized water for 1 hour. For Western blot analysis, proteins were transferred onto PVDF membranes, which were probed with anti-S1 (ABclonal, A20834) or anti-S2 (R&D Systems, MAB10850) rabbit antibodies. S protein and cleavage fragments were imaged using an Odyssey imager and analyzed in ImageStudio (Li-COR Biosciences).

### VSV*ΔG-S_Δ21_ pseudovirus FFU assay.

We used a propagation-competent, chimeric VSV, in which the glycoprotein (G) gene was replaced by a modified SARS-CoV-2 S protein lacking 21 amino acids at the cytosolic C-terminal tail (VSV*ΔG-S_Δ21_) (62). In these experiments, VSV*ΔG-S_Δ21_ titers were determined on Vero/TMPRSS2 cells seeded at 2 × 10^5^ cells/well in 96-well plates 1 day prior to infection. Three experimental setups were used. (i) In pseudovirus preincubation experiments, VSV*ΔG-S_Δ21_ (4 × 10^3^ to 4 × 10^4^ FFU) was treated with purified human CatG, NE, or PR3 at final concentrations of 0.6–5 μg/mL (20–170 nM) for 4 hours at 37°C in PBS without FBS. Then, NSP-treated VSV*ΔG-S_Δ21_ was titrated in DMEM with 10% FBS, which neutralizes the NSPs. (ii) To investigate effects of NSPs on cell surface receptors, cells were washed twice with DMEM (0% FBS) and incubated with NSPs (5 μg/mL) for 1 hour at 37°C, and then washed twice with DMEM with 10% FBS to neutralize proteases prior to infection with 2 × 10^3^ FFU VSV*ΔG-S_Δ21_. (iii) To measure the effects of NSPs on virus adsorbed to cells, 5 × 10^3^ FFU VSV*ΔG-S_Δ21_ was added to Vero/TMPRSS2 for 30 minutes. Unbound pseudovirus was washed and NSPs in DMEM (0% FBS) were added for 4 hours. After 1 hour (i, ii) or 4 hours (iii) in the presence of virus or NSPs, medium was replaced by fresh DMEM with 10% FBS and 1% methylcellulose, and cells were incubated overnight at 37°C and 5% CO_2_. Following aspiration of the supernatant, cells were washed 2 times with PBS, and fixed with formalin (4% v/v). Infected cells were detected by fluorescence microscopy, taking advantage of the virus-encoded GFP reporter, and viral titers were calculated and expressed as FFU/mL.

### NSP-treated-virus infectivity assay.

Vero E6 cells were seeded in 96-well plates 1 day before infection. SARS-CoV-2^614G^ or SARS-CoV-2^MA10^ was pretreated with 5 μg/mL CatG, NE, or PR3 (Athens Research Technology) for 4 hours at 37°C. Briefly, proteases and viral stocks were prediluted in PBS at 0.2 mg/mL and 5 × 10^4^ PFU/mL, respectively. Equal volumes (5 μL) of diluted protease and 5 μL of diluted virus were mixed in 190 μL PBS and incubated for 4 hours at 37°C. The reaction was stopped by addition of DMEM containing 10% FBS and the TCID_50_ assay was performed immediately.

### NSP activity assays.

The proteolytic activity of the human purified NSPs was determined with chromogenic peptide substrates specific for CatG (Ala-Ala-Pro-Phe-pNA) (Sigma-Aldrich) and NE or PR3 (Ala-Ala-Pro-Val-pNA) (Calbiochem). Substrates (250 μg/mL) were incubated at 37°C with NSPs (5 μg/mL) in phenol red–free DMEM with or without 10% FBS in a 96-well plate and absorbance (λ = 405 nm) was measured over time using a GloMax plate reader (Promega).

### Mice.

Mice were housed and bred at the specific pathogen–free mouse facility at the Institute of Virology and Immunology, where they were maintained in individually ventilated cages (blue line, Tecniplast), with 12-hour/12-hour light/dark cycle, 22°C ± 1°C ambient temperature, and 50% ± 5% humidity, autoclaved food, and acidified water. At least 7 days before infection, mice were placed in individual HEPA-filtered cages (IsoCage N, Tecniplast). *Elane^tm1Sds^* (*NE*^–/–^) mice were previously described and backcrossed for 10 generations in the C57BL/6 background (17). *NE*^–/–^ mice were obtained from the Jackson Laboratory (strain 006112), which confirmed by SNP analysis that the mice were in a mixed C57BL6/J and C57BL/6N background. *Ctsg^tm1Ley^* (*CatG*^–/–^) mice were obtained from Christine Pham (Washington University, St. Louis, Missouri, USA), who generated (63) and backcrossed the mice into the C57BL/6J background (64). In our laboratory, *NE*^–/–^ and *CatG*^–/–^ mice were further backcrossed in the C57BL/6J background and were intercrossed to generate homozygous double-deficient *NE.CatG*^–/–^ mice. We report the generation of *Prtn3^em1Cben^* (*PR3*^–/–^) mice by CRISPR/Cas9 ribonucleoprotein complex microinjection in C57BL/6J mouse zygotes at the transgenic facility of the Theodor Kocher Institute, University of Bern. The gRNA targeted sequence was 5′-GCACGAATCTCGGGTGGATC-3′. The resulting allele that we selected included a 2-nucleotide deletion in exon 2 at position 187–188 of the coding sequence, leading to a frameshift and a premature stop codon.

### SARS-CoV-2 infection of mice.

WT, *CatG*^–/–^, *NE*^–/–^, *PR3*^–/–^, and *NE.CatG*^–/–^ male and female mice (8 to 17 weeks old) were anesthetized with isoflurane and inoculated intranasally with 20 μL of SARS-CoV-2^MA10^ per nostril, corresponding to 1 × 10^4^ TCID_50_/mouse. Infected mice were monitored daily for body weight loss and clinical signs of disease. Throat swabs were collected daily under brief isoflurane anesthesia using ultrafine sterile flock swabs (Hydraflock, Puritan, 25-3318-H). The tips of the swabs were incubated with 0.5 mL of RA1 lysis buffer (Macherey-Nagel) supplemented with 1% β-mercaptoethanol and vortexed. Mice were euthanized 2 and 4 dpi. Organs were aseptically dissected, avoiding cross-contamination. The lung right superior lobe and right nasal concha were collected in RA1 lysis buffer and homogenized using a Bullet Blender Tissue Homogenizer (Next-Advance). Homogenates were centrifuged for 3 minutes at 18,000*g* and stored at −70°C until processing for RNA isolation. Lung middle, inferior, and post-caval lobes were collected in 1 mL DMEM and homogenized using M-type tubes and a gentleMACS Tissue Dissociator (Miltenyi Biotec). Samples were centrifuged for 10 minutes at 800*g* and aliquots were stored at –80°C until processing for viral titration and cytokine assay. The lung left lobe was fixed in buffered formalin, embedded in paraffin, and tissue sections were stained with H&E at the COMPATH core facility (University of Bern). Histopathological lung slides were examined and scored by board-certified veterinary pathologists, who were blinded to the identity of the samples and used criteria previously described (65).

### Viral RNA extraction and quantification.

Total RNA extraction from organ homogenates or oropharyngeal swabs was performed with the NucleoMag VET kit (Macherey Nagel) and the KingFisher Flex automated extraction instrument (Thermo Fisher Scientific) following the manufacturers’ instructions. Purity of RNA was assessed using a Nanodrop photometer. Viral copies in organs or swabs were determined using RT-PCR for the viral E gene transcript using the AgPath-ID One-Step RT-PCR (Applied Biosystems) (59). Amplification and detection were performed with the 7500 Real-Time PCR System (Applied Biosystems).

### Viral titration in lung homogenates.

SARS-CoV-2^MA10^ titers in lung homogenates were determined by plaque assay in Vero E6 cells. Confluent cells were inoculated in duplicate with 10-fold serial dilutions of samples in a 24-well plate. The inoculum was removed 1 hour after infection and cells were overlaid with DMEM with 1.2% Avicel (FMC BioPolymer), 15 mM HEPES, 10% FBS, 100 μg/mL streptomycin, and 100 IU/mL penicillin. Plaque-forming units (PFU) were determined after 48 hours.

### Cytokine assay.

Cytokine levels in lung homogenates of mice infected by SARS-CoV-2^MA10^ were measured by the LEGENDplex Mouse Anti-Virus Response Panel (BioLegend, 740621). The data were acquired on a FACSCanto II flow cytometer (BD Biosciences) and analyzed using LEGENDplex software v8.0 (BioLegend).

### Statistics.

Data are presented in violin plots showing individual biological replicates wherever possible, or as mean ± SD or SEM for *n* less than 10 per group or *n* greater than 10 per group, respectively. One- or 2-way ANOVA with Dunnett’s multiple-comparison was used. All statistical analyses were performed using GraphPad Prism (v10). A *P* value of less than 0.05 was considered significant.

### Study approval.

Mouse studies were conducted in compliance with the Swiss Animal Welfare legislation and were approved by the Commission for Animal Experimentation of the Cantonal Veterinary Office of Bern under license number BE43/20.

### Data availability.

The underlying data are available as a Supporting Data Values file in the supplemental material.

## Author contributions

NGFL, AT, and CB designed the project. NGFL, CD, NK, AG, and AT conducted experiments and acquired data. NGFL and NK performed the revision experiments. NGFL, CD, NK, IBV, LGR, AT, and CB analyzed and interpreted the data. MS, PP, NE, GZ, and VT provided key reagents and scientific input. Histopathological lung slides were examined and scored by IBV and LGR. NGFL drafted the manuscript. CB acquired the funding and wrote the manuscript. The manuscript was read and approved by all authors. The order of co-first authors was decided between NGFL, CD, and NK based on their relative contributions after revision of the manuscript.

## Supplementary Material

Supplemental data

Unedited blot and gel images

Supporting data values

## Version 1 03/12/2024

In-Press Preview

## Version 2 04/08/2024

Electronic publication
