# Supplementary material for: Neutrophil proteases are protective against SARS-CoV-2 by degrading the spike protein and dampening virus-mediated inflammation
Source: JCI Insight. 2024 Mar 12;9(7):e174133. doi: 10.1172/jci.insight.174133 (PMC11128203; doi:10.1172/jci.insight.174133)
Supplement: Supplemental data [file jciinsight-9-174133-s102.pdf]

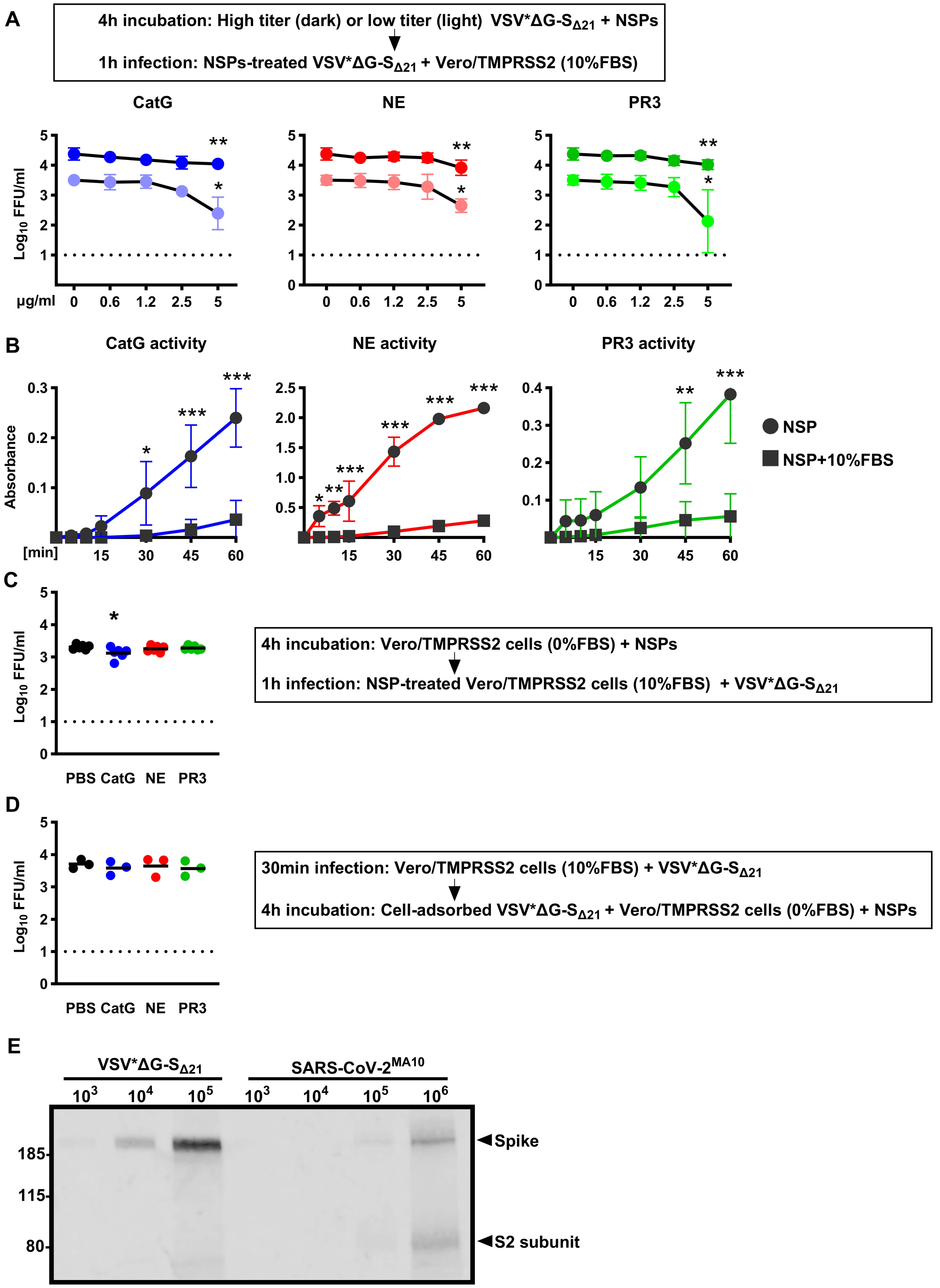
A
4h incubation: High titer (dark) or low titer (light) VSV*ΔG-S$_{Δ21}$ + NSPs
1h infection: NSPs-treated VSV*ΔG-S$_{Δ21}$ + Vero/TMPRSS2 (10%FBS)
CatG
NE
PR3
Log$_{10}$ FFU/ml
µg/ml
0 0.6 1.2 2.5 5
B
CatG activity
NE activity
PR3 activity
Absorbance
[min]
15 30 45 60
NSP
NSP+10%FBS
C
Log$_{10}$ FFU/ml
PBS CatG NE PR3
4h incubation: Vero/TMPRSS2 cells (0%FBS) + NSPs
1h infection: NSP-treated Vero/TMPRSS2 cells (10%FBS) + VSV*ΔG-S$_{Δ21}$
D
Log$_{10}$ FFU/ml
PBS CatG NE PR3
30min infection: Vero/TMPRSS2 cells (10%FBS) + VSV*ΔG-S$_{Δ21}$
4h incubation: Cell-adsorbed VSV*ΔG-S$_{Δ21}$ + Vero/TMPRSS2 cells (0%FBS) + NSPs
E
VSV*ΔG-S$_{Δ21}$
SARS-CoV-2^{MA10}
10^3 10^4 10^5 10^3 10^4 10^5 10^6
185
115
80
Spike
S2 subunit

Supplemental Figure 1. Effects of NSPs on entry of VSV*ΔG-$S_{Δ21}$ in Vero/TMPRSS2 cells. (**A**) VSV*ΔG-$S_{Δ21}$ (4x10^4 FFU dark dots; or 4x10^3 FFU light dots) were preincubated with purified NSPs (0.6-5µg/ml) for 4h at 37°C in PBS. Vero/TMPRSS2 cells in DMEM containing 10% FBS were then inoculated with serial dilutions of NSP-treated VSV*ΔG-$S_{Δ21}$ to determine titers. Data are shown as mean ± SD of 4 independent experiments and were analyzed by one-way ANOVA with Dunnett's multiple comparisons test, comparing the NSP-treated groups to the PBS control; * *P*<0.05, ** *P*<0.01. (**B**) Activity of NSPs (5µg/ml) in phenol red-free DMEM containing 0% or 10% FBS was determined using specific chromogenic substrates for CatG (Ala-Ala-Pro-Phe), NE or PR3 (Ala-Ala-Pro-Val). Data represent mean ± SD and were analyzed by two-way ANOVA with Sidak's multiple comparisons test, comparing the effect of serum at each time point (n=3); * *P*<0.05, ** *P*<0.01, *** *P*<0.001. (**C**) Vero/TMPRSS2 cells were incubated with NSPs (5µg/mL) for 4h in FBS-free DMEM. Cells were inoculated with 4x10^3 FFU VSV*ΔG-$S_{Δ21}$ for 1h in DMEM with 10% FBS. Data were analyzed by one-way ANOVA with Dunnett's multiple comparisons test, comparing the NSP-treated groups to the control (n=6); * *P*<0.05. (**D**) Titers of cell-adsorbed VSV*ΔG-$S_{Δ21}$ incubated with NSPs. Vero/TMPRSS2 cells were inoculated with 4x10^3 FFU VSV*ΔG-$S_{Δ21}$ for 30 minutes at 37°C, 5% CO_2 allowing virus cell adsorption. Then, medium was removed and FBS-free medium containing purified human CatG, NE, or PR3 at a final concentration of 5 µg/ml was added. Vero/TMPRSS2 cells, cell adsorbed- VSV*ΔG-$S_{Δ21}$ and NSPs were incubated for 4h at 37°C, 5% CO_2. Data were analyzed by one-way ANOVA with Dunett's multiple comparisons test, comparing the NSP-treated groups to the PBS control (n=3). (**A-D**) Infected cells were detected by fluorescence microscopy and virus titers were calculated and expressed as FFU/ml. (**E**) Immunoblot of VSV*ΔG-$S_{Δ21}$ and SARS-CoV-2^{MA10} lysates probed with anti-S2 subunit antibody.
