# Supplementary material for: Neutrophil proteases are protective against SARS-CoV-2 by degrading the spike protein and dampening virus-mediated inflammation
Source: JCI Insight. 2024 Mar 12;9(7):e174133. doi: 10.1172/jci.insight.174133 (PMC11128203; doi:10.1172/jci.insight.174133)

Full unedited gels for figure 1.A

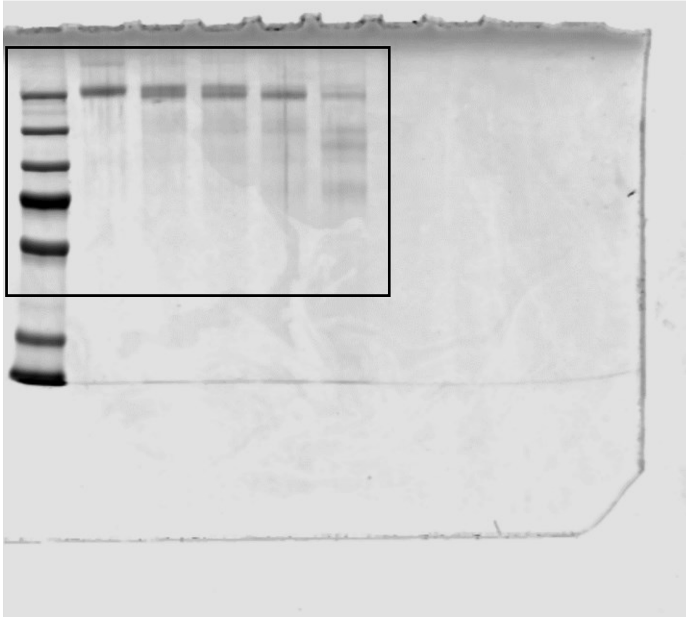

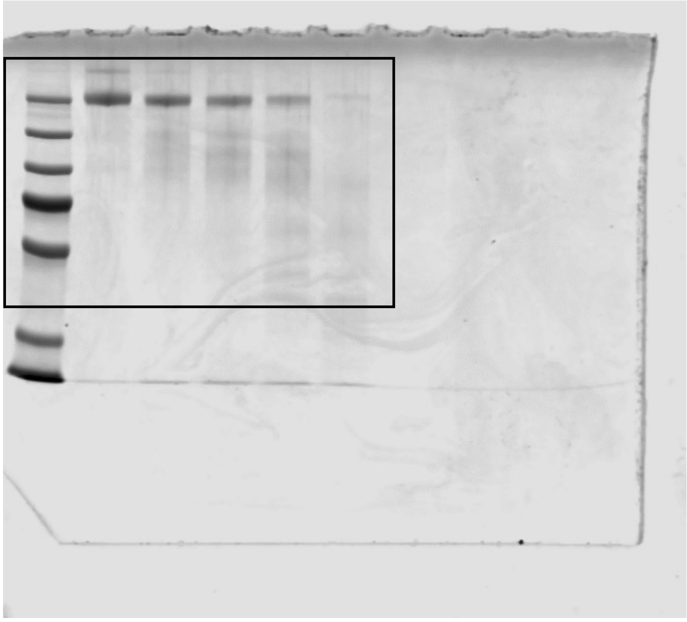

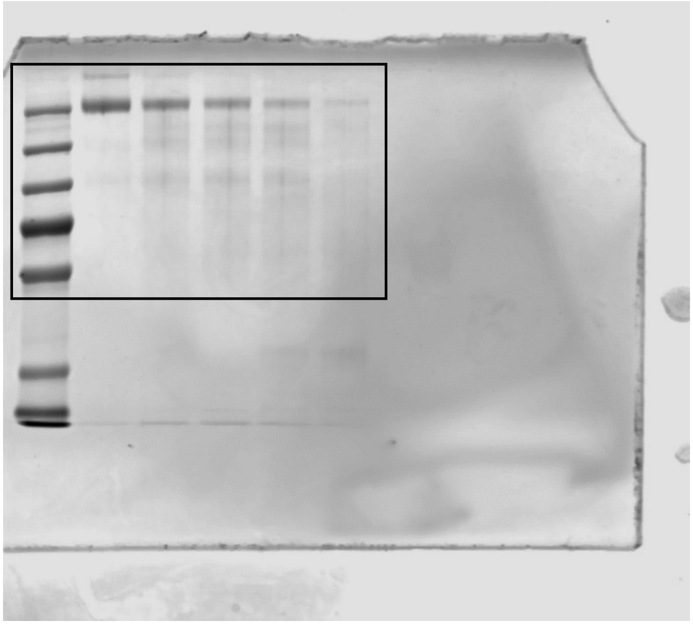

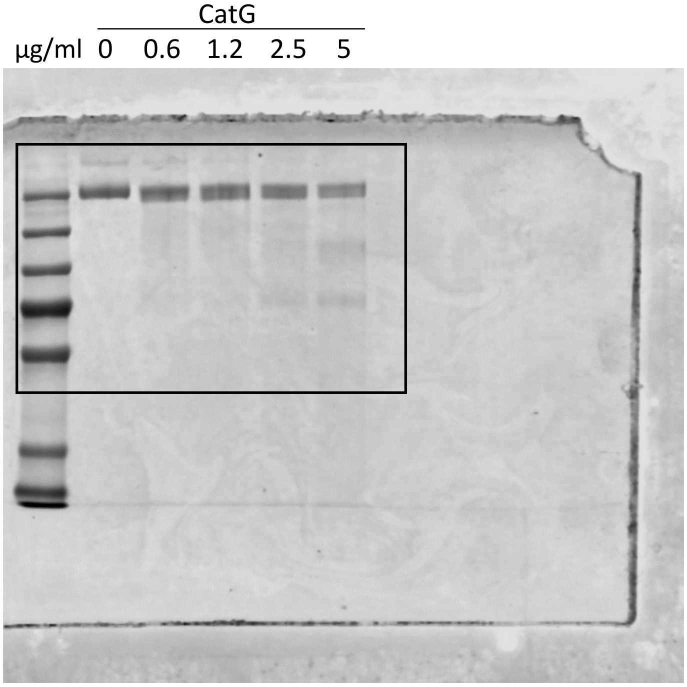

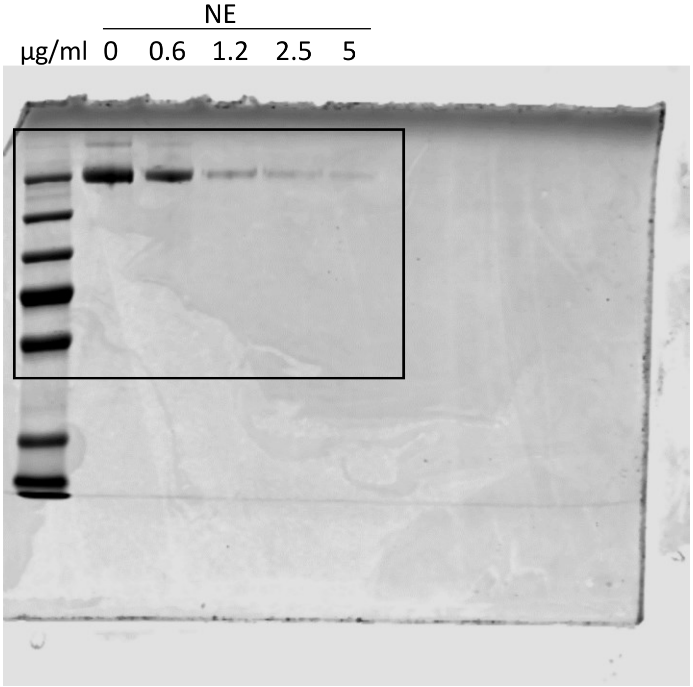

Full unedited gels
for figure 1.B

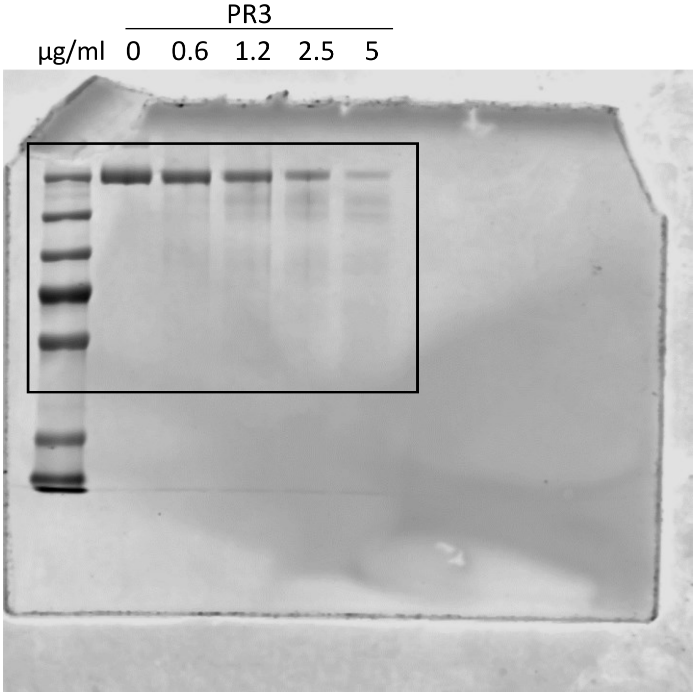

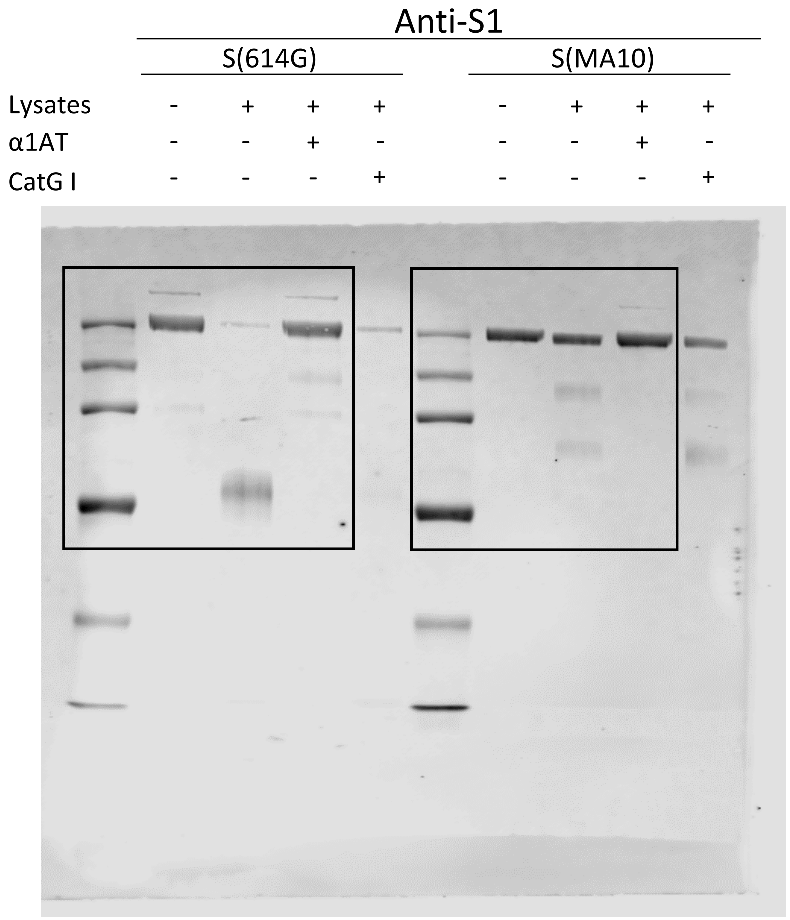

Full unedited blot for figure 1.C

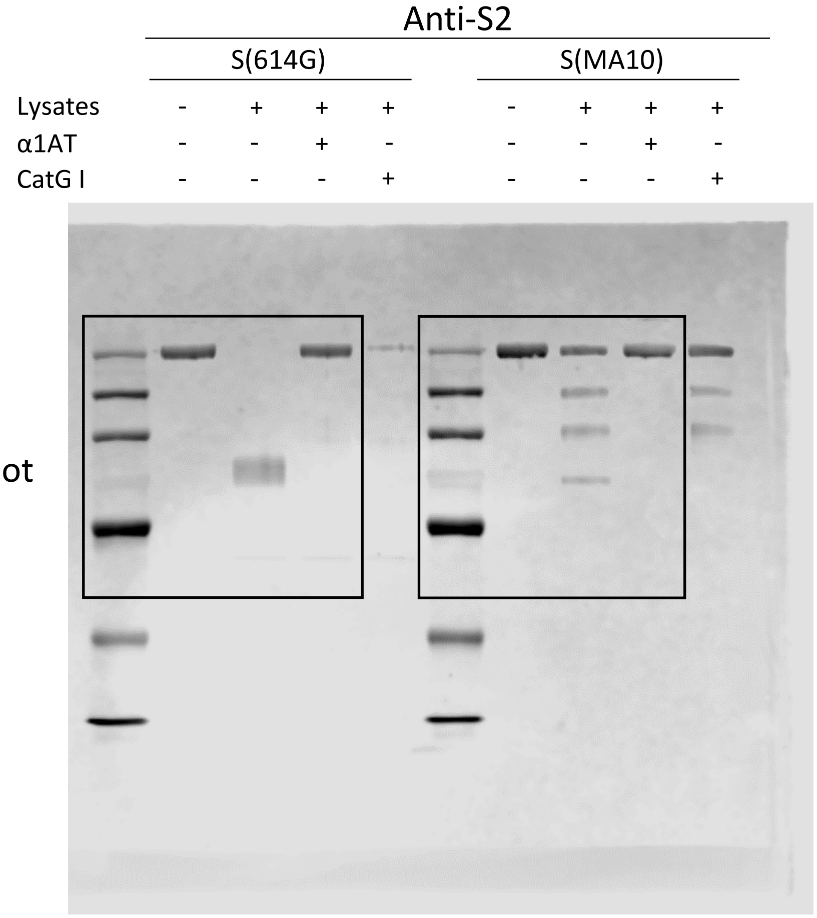

Full unedited blot for figure 1.D

Full unedited blot for supplementary figure 1.E

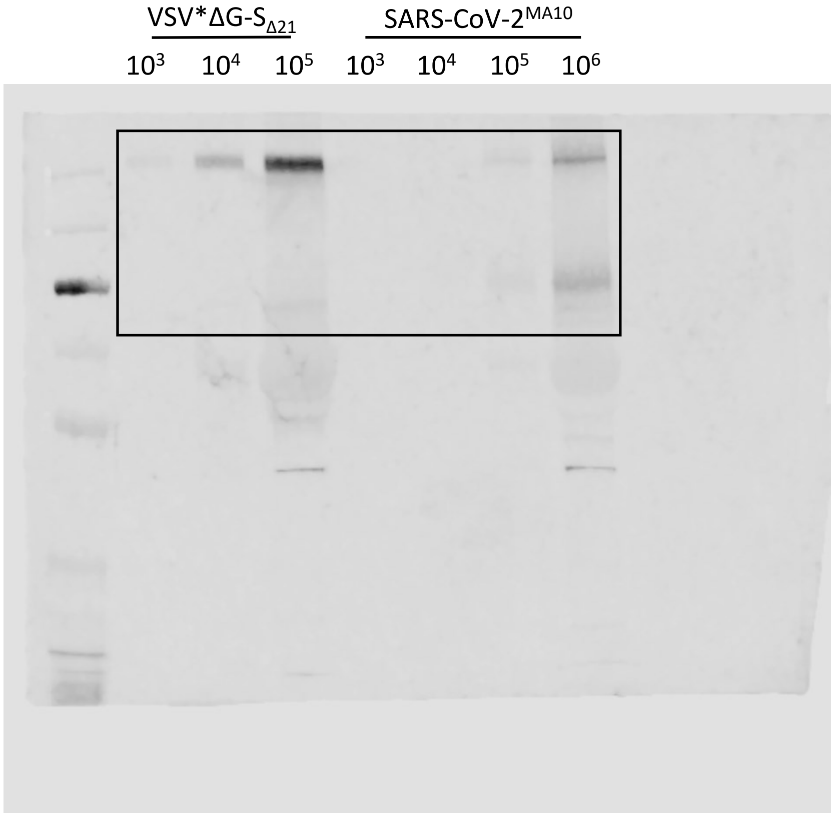

Supplement: Unedited blot and gel images [file jciinsight-9-174133-s103.pdf]
